# Prevalence of Ear-Piercing Complications, Associated Risk Factors, and Current Ear-Piercing Trends Among Females in Riyadh, Saudi Arabia

**DOI:** 10.3390/healthcare14152265

**Published:** 2026-07-24

**Authors:** Mayar Abdulsalam Alsaqr, Najlaa Abdulrahman Alsubeeh, Saleh Alabood, Fatima Saad Alangari, Subhi M. K. Zino Alarki, Laila Salah Aldokhail, Raghad Abdulhai Ghazzawi, Mohammed Alkarzae, Badi Aldosari

**Affiliations:** 1 Department of Otolaryngology–Head & Neck Surgery, King Saud University Medical City, King Saud University, Riyadh 12372, Saudi Arabia; fatimaalangari@hotmail.com (F.S.A.); subhi.k.z@gmail.com (S.M.K.Z.A.); 2 Ministry of Health Headquarters, Riyadh 12233, Saudi Arabia; najlaa.alsubeeh@gmail.com; 3 Otolaryngology–Head & Neck Surgery Unit, Surgery Department, Specialized Medical Center, Riyadh 12311, Saudi Arabia; saleh.alabood@hotmail.com; 4 College of Medicine, Princess Norah Bint Abdulrahman University, Riyadh 11564, Saudi Arabia; aldokhail.laila@gmail.com; 5 Department of Otolaryngology–Head and Neck Surgery, King Faisal Specialist Hospital and Research Centre, Jeddah 23433, Saudi Arabia; raghadagh@hotmail.com; 6 Otorhinolaryngology–Head and Neck Surgery, Security Forces Hospital, Riyadh 12625, Saudi Arabia; m.alkarzae@hotmail.com; 7 Facial Plastic Division, ENT Department, King Abdulaziz University Hospital, Riyadh 12629, Saudi Arabia; baaldosari@ksu.edu.sa

**Keywords:** ear piercing, complications, body piercing, infection, risk factors, cartilage piercing, public health

## Abstract

**Highlights:**

**What are the main findings?**

- More than one-quarter of females reported experiencing ear-piercing complications, with infection and pain being the most frequently reported adverse outcomes.
- Cartilage piercing, multiple piercings, student status, and the use of piercing guns were identified as independent predictors of complications.

**What are the implications of the main findings?**

- Public awareness regarding ear-piercing risks and aftercare remains limited, highlighting the need for improved education and counseling before the procedure.
- Promoting safer piercing techniques and reducing exposure to modifiable risk factors may help decrease the burden of ear-piercing complications.

**Abstract:**

Background: Ear piercing is one of the most commonly performed forms of body modification worldwide. While generally considered a simple procedure, it may be associated with a range of local and cosmetic complications. Objective: To evaluate current ear-piercing trends, determine the prevalence of ear-piercing complications, and identify factors independently associated with the development of these complications among females residing in Riyadh. Methods: A cross-sectional survey was conducted among females residing in Riyadh, Saudi Arabia. Participants were recruited through an online self-administered questionnaire distributed via social media platforms. Multivariable logistic regression was used to identify independent predictors of complications. Results: A total of 1018 participants were included. Ear piercing for aesthetic purposes was reported by 52.8% of respondents. Most participants (87.8%) reported not receiving information regarding procedural risks or aftercare instructions. Complications were reported by 26.5% of participants, with infection (57.0%) and ear pain (54.1%) being the most frequent. Independent predictors of complications included having multiple piercings, cartilage piercing, student status, and the use of piercing guns. Undergoing the first ear piercing before one year of age was associated with a lower risk of complications. Conclusions: Our findings indicate that nearly one-quarter of females reported experiencing complications from ear piercings and are associated with several potentially modifiable factors. This emphasizes the importance of informed consent before the procedure, along with proper education on associated risks.

## 1. Introduction

Ear piercing is among the most frequently performed esthetic procedure worldwide and it represents one of the oldest forms of body modification practiced across different cultures and age groups. Although traditionally limited to the earlobe, contemporary ear-piercing trends increasingly involve cartilaginous sites such as the helix, tragus, antihelix, and scapha, reflecting evolving esthetic preferences and fashion influences [1,2,3,4]. Despite its widespread acceptance and generally favorable safety profile, ear piercing is not without risk and may result in a variety of adverse outcomes.

Reported complications range from minor localized reactions to more serious conditions requiring medical intervention. Commonly described complications include infection, pain, bleeding, allergic contact dermatitis, embedded earrings, hypertrophic scars, keloids, split earlobes, and cartilage deformities [1,4,5,6,7,8].

Several factors have been proposed to influence the likelihood of developing ear-piercing complications. These include the anatomical location of the piercing, the technique used, the type of jewelry material, procedural sterility, practitioner experience, and compliance with aftercare recommendations [5,9,10,11]. Cartilage piercings have attracted particular attention because the relatively limited vascularity of auricular cartilage may predispose individuals to delayed healing and infectious complications, including perichondritis and chondritis [6,7,8]. Similarly, concerns have been raised regarding the use of piercing guns because of their potential association with tissue trauma and sterilization challenges [10,11,12,13].

Despite the growing body of international evidence evaluating ear-piercing complications and associated risk factors, contemporary epidemiological data on ear-piercing practices, complications, and associated risk factors in Saudi Arabia remain scarce. This lack of local evidence limits the ability to accurately characterize the burden of ear-piercing complications within the Saudi population and to develop evidence-based recommendations tailored to local clinical practice and public health needs. Although ear piercing is generally considered as a minor cosmetic procedure, its complications may require medical evaluation, antibiotic therapy, or surgical intervention in severe cases, representing a preventable healthcare burden [5]. While several international studies have examined ear-piercing practices, complication prevalence, and associated risk factors [3,4], comparable data from Saudi Arabia remain limited.

Therefore, this study aimed to evaluate current ear-piercing trends among females in Riyadh, determine the prevalence of ear-piercing complications, and identify factors independently associated with the development of these complications.

## 2. Materials and Methods

### 2.1. Study Design, Setting, Sample Size, and Participant Recruitment

A cross-sectional survey was conducted in Riyadh, Saudi Arabia, over a 5 months period (August–December 2025). The minimum required sample size for this cross-sectional study was calculated using a 95% confidence level and a 5% margin of error, yielding a minimum sample size of 385 participants. We received 2063 complete responses. Since the number of eligible questionnaires exceeded the required sample size based on our a priori sample size calculation, a simple random sample was obtained, and a final 1018 responses were included in the analysis to maintain representativeness of the responses and minimize selection bias.

Participants were recruited through the public distribution of an anonymous Arabic-language online questionnaire disseminated via social media platforms. This recruitment strategy was selected because of the high level of internet accessibility and social media utilization among residents of Riyadh. According to the General Authority for Statistics, 86.8% of Riyadh residents had internet access, and 73.3% used the internet primarily for social networking and related platforms [14].

Eligible participants were females residing in Riyadh who had undergone at least one ear-piercing procedure. Responses from participants residing outside Riyadh, male respondents, individuals without a history of ear piercing, and questionnaires that did not meet the predefined eligibility criteria were excluded.

### 2.2. Questionnaire Development and Data Collection

The questionnaire was developed by the research team after an extensive review of the relevant literature on ear-piercing practices and complications. It was designed in Arabic to ensure accessibility for the target population and was administered electronically. The survey link was distributed through social media platforms. The questionnaire consisted primarily of close-ended multiple-choice questions and comprised 20 questions organized into three sections. The first section included six questions on participant demographics (age, nationality, educational level, marital status, occupational status, and monthly family income). The second section contained nine questions on ear-piercing characteristics and procedural practices, including age at first ear piercing, number of ear piercings, anatomical piercing sites, piercing methods, jewelry materials, reasons for piercing, practitioner type, informed consent practices, and provision of aftercare instructions and risk counseling. The third section consisted of five questions addressing post-piercing complications, including the occurrence and type of complications, the anatomical site involved, healthcare-seeking behavior, self-management practices, and hospitalization related to ear-piercing complications. Multiple responses were permitted for questions regarding piercing sites, piercing methods, reasons for piercing, practitioner type, complication types, affected anatomical sites, and management practices.

Before distribution, the questionnaire underwent face and content validation by two facial plastic surgery consultants with experience in clinical research to assess its clarity, relevance, and comprehensiveness. Subsequently, a pilot study was conducted on 30 participants to evaluate the questionnaire’s readability, comprehensibility, flow, and average completion time (approximately 5 min). Participants included in the pilot study were excluded from the final analysis.

### 2.3. Data Quality Assurance

All submitted questionnaires were screened before analysis to ensure data quality and participant eligibility. Responses were reviewed for completeness, duplication, internal consistency, and compliance with the predefined inclusion and exclusion criteria. Duplicate submissions were identified using response patterns and IP addresses. Questionnaires from ineligible participants, duplicate entries, and responses containing missing or inconsistent data were excluded. IP addresses were used solely for data-quality screening and were permanently deleted after completion of the data-cleaning process to preserve participant confidentiality. The number of questionnaires excluded at each stage and the corresponding reasons are presented in the participant flow diagram.

### 2.4. Ethical Considerations

The study adhered to internationally accepted ethical principles of the Declaration of Helsinki. Electronic informed consent was obtained from all participants before survey initiation. Participation was voluntary, and participants were free to discontinue the survey at any time. No financial incentives or rewards were provided.

To maintain confidentiality, no identifying personal information was collected, and all responses were analyzed anonymously. Access to the data was restricted to the research team.

### 2.5. Statistical Analysis

Data analysis was conducted using the Statistical Package for the Social Sciences (SPSS), version 23 (IBM Corp., Armonk, NY, USA). Categorical variables were summarized using frequencies and percentages. Associations between categorical variables were assessed using the chi-square test. Univariate logistic regression analysis was initially performed to evaluate the association between potential risk factors and the occurrence of ear-piercing complications. Variables considered statistically significant in the univariate analysis were subsequently entered into a multivariable logistic regression model to identify independent predictors of complications. Regression coefficients (B), odds ratios (ORs), corresponding 95% confidence intervals (CIs), and *p*-values were reported. A two-sided *p*-value < 0.05 was considered statistically significant.

## 3. Results

### 3.1. Sociodemographic and Participant Characteristics

1018 questionnaires were included in the final analysis. Participants were categorized into four age groups, with the largest proportion being older than 40 years (*n* = 374, 36.7%). Most participants were Saudi nationals (*n* = 988, 97.0%), had a university-level education or higher (*n* = 717, 70.4%), and were married (*n* = 564, 55.4%). Regarding occupational status, 399 (39.2%) were unemployed, 319 (31.3%) were students, and 300 (29.5%) were employed (Table 1).

### 3.2. Ear-Piercing Trends and Influencing Factors

The majority of participants (*n* = 757, 74.4%) underwent their first ear piercing before one year of age, and 567 (55.7%) had more than one ear piercing. The earlobe was the most commonly pierced site (*n* = 985, 96.8%), followed by the helix (*n* = 294, 28.9%) and the scapha (*n* = 187, 18.4%). Piercing guns were the most frequently used method (*n* = 695, 68.3%), gold was the most commonly worn jewelry material immediately after piercing (*n* = 559, 54.9%), and most procedures were performed by medical practitioners (*n* = 673, 66.1%). Cosmetic appearance was the most common reason for ear piercing (*n* = 538, 52.8%). Most participants reported that they had not received written informed consent before the procedure (*n* = 827, 81.2%) or counseling regarding ear-piercing risks and aftercare (*n* = 894, 87.8%) (Table 2).

**Table 2:** Current Ear-Piercing Trends and Influencing Factors.

Variable	Frequency	%
How old were you when you had your first ear piercing?		
1–5 years old	166	16.3%
11 years old and older	25	2.4%
6–10 years old	70	6.9%
Younger than 1 year	757	74.4%
How many ear-piercings do you have?		
1	451	44.3%
2	302	29.7%
3	133	13.1%
4	64	6.3%
5	22	2.1%
More than 5	46	4.5%
Ear-piercing sites (Figure 1)		
(1) Lobule	985	96.8%
Other sites:		
(2) Helix	294	28.9%
(3) Scapha	187	18.4%
(4) Antihelix	14	1.4%
(5) Inferior crus of antihelix	4	0.4%
(6) Concha	8	0.8%
(7) Helix crus	6	0.6%
(8) Tragus	33	3.2%
(9) Antitragus	4	0.4%
Methods of ear-piercing?		
Piercing gun	695	68.3%
Others:		
Needle and thread	394	38.7%
Earring studs	95	9.3%
Material of the earrings worn after piercing?		
Gold	559	54.9%
I don’t remember/not sure	287	28.2%
Metal/Stainless steel	65	6.4%
Plastic	10	1.0%
Silver	97	9.5%
Reasons for having ear-piercing?		
Beauty	538	52.8%
Other reasons:		
Influenced by friends	51	5%
as fashion trend	191	18.8%
willingness for a new experiment	131	12.9%
gender identification	324	31.8%
way to express adolescence	3	0.3%
Way to express pain tolerance	7	0.7%
Culture	171	16.8%
To record a special event.	10	1.0%
Who did your ear-piercing?		
Medical practitioner	673	66.1%
Others:		
Mobile piercer/Home-based esthetician	52	5.1%
It was personally done	55	5.4%
Piercing shop specialist	125	12.3%
Friend or relative	322	31.6%
Was written informed consent obtained before ear piercing?		
No	827	81.2%
Yes	191	18.8%
Did the practitioner explain to you ear-piercing related risks and maintenance instructions?		
No	894	87.8%
Yes	124	12.2%

Multiple answers were allowed for piercing sites, piercing methods, reasons for piercing, and practitioner type.

**Figure 1:**
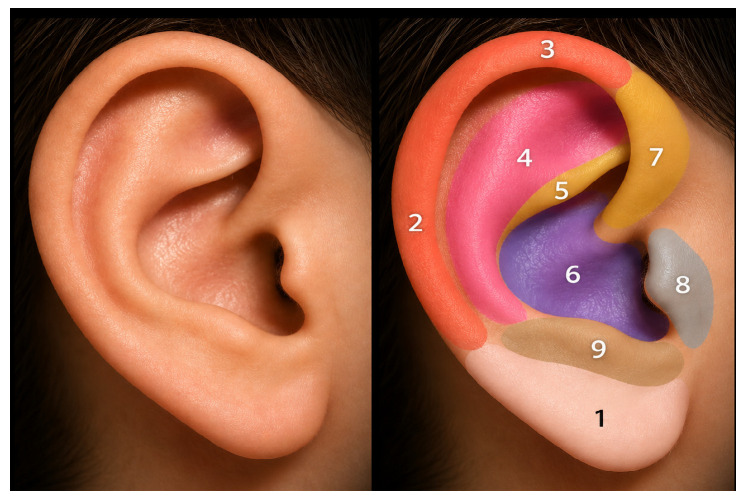
Anatomical ear-piercing sites presented in the questionnaire, with participants selecting their piercing site based on the numbered locations shown.

### 3.3. Prevalence and Characteristics of Ear-Piercing Complications

Overall, 270 participants (26.5%) reported experiencing at least one ear-piercing complication, whereas 748 (73.5%) reported no complications. The most commonly reported complications were Infection (*n* = 154, 57.0%) and ear pain (*n* = 146, 54.1%). The earlobe was the most frequently affected site (*n* = 142, 52.6%), followed by the helix (*n* = 105, 38.9%). Most participants managed complications through self-medication (*n* = 158, 58.5%), while only 1.5% required hospital admission (Table 3).

### 3.4. Factors Associated with Ear-Piercing Complications

Multivariable logistic regression identified several independent predictors of ear-piercing complications (Table 4). Having multiple piercings (*p* < 0.001), student status (*p* = 0.031), cartilage piercing (*p* = 0.004), and the use of piercing guns (*p* = 0.015) were significantly associated with an increased risk of complications. In contrast, undergoing the first ear piercing before one year of age was associated with a lower risk of complications (*p* = 0.009). Overall, ear-piercing complications were reported by approximately one quarter of participants and were independently associated with several modifiable procedural and participant related factors, including multiple piercings, cartilage piercing, and the use of piercing guns.

## 4. Discussion

In this cross-sectional survey, 26.5% of respondents reported experiencing at least one ear-piercing complication, with infection and pain being the most commonly reported complications. Several independent predictors of complications were identified, including student status, undergoing the first ear piercing after one year of age, having multiple piercings, cartilage piercing, and the use of piercing guns. These findings contribute to the limited epidemiological evidence regarding ear-piercing complications in Saudi Arabia and identify several potentially modifiable risk factors that may help inform preventive strategies and patient education.

The prevalence of complications observed in the present study is consistent with previously reported rates ranging from 20% to 35% in the literature [1,2]. Despite ear piercing being widely perceived as a simple cosmetic procedure, these findings demonstrate that complications remain relatively common and may represent an underrecognized public health concern.

Anatomically, the ear is largely made of elastic cartilage which gives it its shape, except the lobule which consists of fibro fatty tissue [3,15]. Ear piercings can be performed at multiple anatomical sites, including the earlobe, helix, antihelix, concha, tragus, antitragus, and scapha [3,4,5]. A major finding of this study was the significant association between cartilage piercing and complications. Participants with cartilage piercings were more likely to report adverse outcomes than those without cartilage involvement. This association is biologically plausible, as auricular cartilage has a relatively limited vascular supply, predisposing it to delayed healing and increased susceptibility to infection [6,7,8,9]. Moreover, cartilage lacks an intrinsic blood supply and relies on the perichondrium for nutrition. Consequently, once bacteria are introduced during piercing, local host defense is reduced, and infections may progress rapidly. The avascular nature of cartilage also limits the effectiveness of systemic antibiotics after infection is established, increasing the risk of cartilage necrosis and permanent deformity if treatment is delayed [1,16]. serious complications such as perichondritis and chondritis have been reported following cartilage piercing and may result in permanent cosmetic deformity if not managed appropriately [6]. This supports the biological plausibility of our findings and highlights cartilage piercing as a high-risk practice. In contrast, Simplot and Hoffman, in a study conducted in the United States among individuals with cartilage and soft-tissue ear piercings, found no statistically significant difference in complication rates between the two groups.

The use of piercing guns was also identified as an independent risk factor for complications. Previous studies have suggested that piercing guns may increase tissue trauma and present challenges regarding sterilization compared with alternative piercing techniques [5,7,8,10,11]. In addition, inadequate sterilization of reusable piercing devices may increase the risk of bacterial and blood-borne infection transmission [12]. However, not all studies have reported consistent findings. One study found that the risk of perichondritis was not significantly influenced by the piercing technique itself and suggested that aseptic technique, proper sterilization, cleanliness, and appropriate post-piercing care may have a greater impact on clinical outcomes than the piercing method alone [13]. Taken together, these findings suggest that both the choice of piercing technique and adherence to appropriate infection-control and aftercare practices play important roles in reducing the risk of complications.

Another important finding was the association between multiple piercings and complication occurrence. Individuals with more than one piercing were significantly more likely to experience complications, which may reflect increased cumulative exposure to tissue injury and a greater likelihood of undergoing higher-risk piercing procedures. Similarly, Armstrong et al. in a study of college students in the United States, reported that individuals with multiple body piercings are at a higher risk of both local and systemic complications [17].

Interestingly, participants who underwent their first ear piercing before one year of age demonstrated a lower risk of complications. Although this association should be interpreted within the context of the cross-sectional study design, previous studies have suggested that earlier ear piercing may be associated with improved healing and a lower risk of certain complications. Gabriel et al., in a cross-sectional study of the Nigerian population, reported that many parents chose to pierce their children’s ears during the first week of life because they believed that it was associated with reduced pain while the tissue remained soft, easier cooperation, and faster healing, which may contribute to a lower risk of complications [3]. Moreover, Lane et al. suggested that if ear piercing is desired in a family that is prone to developing keloids, it is better for them to have their children’s ears pierced during early childhood [18].

An interesting finding in the present study was that most ear piercings were performed by medical practitioners (66.1%). Previous studies have reported that ear piercing is more commonly performed by non-medical practitioners, including friends, relatives, and piercing-shop personnel [5,9,19,20]. The higher proportion of medically performed piercings observed in our study may reflect differences in local healthcare accessibility, cultural practices, or parental preferences, particularly given that most participants underwent their first ear piercing during infancy. Nevertheless, practitioner type was not independently associated with complication occurrence after adjustment for other factors.

Student status was identified as an independent predictor of ear-piercing complications. Although the underlying explanation remains uncertain, this finding may reflect behavioral and social factors, including a greater likelihood of having multiple piercings, increased influence of peer-related trends, or engagement in higher-risk piercing practices. Similar observations have been reported in studies involving younger populations, where body modification practices were associated with a higher frequency of adverse outcomes [19].

A concerning finding in this study is the limited provision of informed consent and proper patient education. More than four-fifths of participants reported not receiving information regarding procedural risks or maintenance instructions before piercing. Although this variable was not independently associated with complications after adjustment for confounding factors, its clinical importance should not be underestimated. Improved patient education may facilitate early recognition of complications and promote safer piercing practices [12,19].

From a public health perspective, these findings highlight the importance of promoting safe ear-piercing practices and increasing awareness regarding potential complications. Educational initiatives targeting individuals considering ear piercing, as well as practitioners performing the procedure, may help reduce complication rates. Particular attention should be given to cartilage piercings and the use of piercing guns because of their association with adverse outcomes. Establishing standardized recommendations for piercing techniques, infection prevention measures, and aftercare counseling may further improve safety and reduce the burden of preventable complications.

This study has several strengths, including its relatively large sample size, comprehensive assessment of piercing-related characteristics, and use of multivariable regression analysis to identify independent predictors of complications. These strengths provide a broad overview of contemporary ear-piercing practices among females in Riyadh and contribute valuable local epidemiological data to a field in which evidence from Saudi Arabia remains limited. Nevertheless, some limitations should be considered when interpreting the findings. The cross-sectional design does not permit causal inferences, and complication data were based on participant reports rather than clinical confirmation. In addition, because many participants underwent their first ear piercing during early childhood, some degree of recall bias may have occurred. Despite these limitations, this study provides important epidemiological evidence regarding ear-piercing complications in Saudi Arabia and identifies several potentially modifiable risk factors that may inform preventive strategies, patient counseling, and future research. Future research should focus on prospective studies to better establish causal relationships and evaluate the effectiveness of preventive strategies, including standardized education protocols and regulation of piercing practices, and further investigate the long-term impact of the age at first ear piercing on subsequent piercing behaviors and complication risk.

## 5. Conclusions

This study demonstrates that ear-piercing complications are relatively common, affecting approximately one in four individuals. Infection remains the most frequently reported complication, and several modifiable risk factors were identified, including cartilage piercing, multiple piercings, and the use of piercing guns. Improved counseling, informed consent practices, and evidence-based aftercare education may contribute to reducing complication rates and improving patient outcomes.

## Figures and Tables

**Table 1:** Sociodemographic and Participant Characteristics.

Variable	*N*	%
Age:		
10 to 19	165	16.2%
20 to 29	234	23%
30 to 39	245	24.1%
more than 40	374	36.7%
Nationality:		
Non-Saudi	30	3%
Saudi	988	97%
Educational level:		
Secondary or less	301	29.6%
University or above	717	70.4%
Marital status:		
Single	454	44.6%
Married	564	55.4%
Occupational status:		
Student	319	31.3%
Unemployed	399	39.2%
Employed	300	29.5%
Family’s monthly income:		
<11,000 SAR	348	34.2%
11,000–15,000 SAR	225	22.1%
16,000–25,000 SAR	237	23.3%
≥26,000 SAR	208	20.4%

**Table 3:** Prevalence of ear-piercing complications.

Variable	Frequency	%
Have you experienced any complications after ear piercing?		
No	748	73.5%
Yes	270	26.5%
What complications have you experienced?		
Keloid/Hypertrophic scar	14	5.2%
Ear deformity/Cauliflower	8	3.0%
Ear bleeding	58	21.5%
Ear pain	146	54.1%
Embedded earrings	46	17.0%
Allergic reaction	58	21.5%
Ear split	10	3.7%
Infection	154	57.0%
What is the piercing site that was affected by the previous complication?		
Lobule	142	52.6%
Other sites:		
Helix	105	38.9%
Scapha	70	25.9%
Anti helix	3	1.1%
inferior crus of anti-helix	1	0.4%
Concha	0	0
Helix crus	3	1.1%
Tragus	8	3.0%
Antitragus	2	0.7%
How did you react to the previous complication?		
I didn’t do anything	25	9.3%
Went to or contacted the piercer	13	4.8%
Took a pain killer	50	18.5%
Self-medicated	158	58.5%
Went to the Emergency Department	5	1.9%
Went to a physician	32	11.9%
Took a pharmacist advice	26	9.6%
alternative medicine	37	13.7%
Have you been admitted to the hospital for that complication?		
No	266	98.5%
Yes	4	1.5%

Percentages for complication types, anatomical sites, and management practices were calculated based on participants who reported complications (*N* = 270). Multiple responses were allowed.

**Table 4:** Multivariate logistic regression for risk factors of having ear piercing complications.

Possible Risk Factors	Multivariate Logistic Regression				
	B	OR	95% CI		*p*-Value
			Lower	Upper	
Occupational status: **					0.022
student	0.518	1.68	1.047	2.688	0.031
Unemployed	−0.160	0.85	0.569	1.276	0.439
Employed *		1.00			
How old were you when you had your first ear piercing? **					
younger than1 year old	−1.21	0.30	0.12	0.74	0.009
>1 year old *		1.00			
Number of piercings **					
>1	1.082	2.95	1.887	4.611	<0.001
1 *		1.00			
Lobule piercing site **					
No	1.298	3.66	1.646	8.153	0.001
Yes *		1.00			
Cartilage piercing site **					
No	−0.534	0.59	0.406	0.846	0.004
Yes *		1.00			
Method of piercing **					
Others (Needle and thread/earring studs)	−0.555	0.57	0.367	0.899	0.015
Piercing gun *		1.00			
The material of the earrings was worn after the piercing					
Others (Metal-Stainless steel/Plastic/Silver)	0.394	1.48	0.945	2.328	0.086
Gold *		1.00			
Done by whom?					
Others	−0.198	0.82	0.560	1.202	0.310
Medical practitioner *		1.00			
Any explanation was provided regarding ear-piercing related risks and maintenance instructions?					
No	−0.357	0.70	0.450	1.088	0.113
Yes *		1.00			

CI: confidence interval. Reference categories are indicated by (*). Statistically significant results were defined as *p* < 0.05 and indicated by (**).

## Data Availability

The data presented in this study are available from the corresponding author upon reasonable request. The data are not publicly available due to privacy and ethical restrictions.
